# Covert cerebrospinal fluid dynamics dysfunction: evolution from conventional to innovative therapies

**DOI:** 10.3389/fneur.2025.1554813

**Published:** 2025-03-12

**Authors:** Yi Xu, Hua Yin, Lingge Li, Xiaodi Wang, Qinghua Hou

**Affiliations:** ^1^Department of Rehabilitation Medicine, The Seventh Affiliated Hospital, Sun Yat-sen University, Shenzhen, China; ^2^Class 6, 2020 Clinical Medicine Program, Sun Yat-Sen University, Shenzhen, China; ^3^Class 2, 2020 Clinical Medicine Program, Sun Yat-Sen University, Shenzhen, China; ^4^Department of Neurology, Clinical Neuroscience Center, The Seventh Affiliated Hospital, Sun Yat-sen University, Shenzhen, China

**Keywords:** cerebrospinal fluid, glymphatic, lymphatics, neuromodulation, therapy

## Abstract

Cerebrospinal fluid (CSF) dynamics disorders are intricately linked to diverse neurological pathologies, though they usually are mild and covert. Contemporary insights into glymphatic system function, particularly the CSF transport, drainage, and its role in clearing metabolic waste and toxic substances in both normal and pathological states, and the pivotal role of aquaporin-4 (AQP4) in CSF-interstitial fluid (ISF) exchange, have established novel theoretical frameworks of subclinical CSF dynamics dysfunction, and have promoted the development of non-surgical therapeutic approaches for them simultaneously. This review comprehensively analyzes the advancement of non-surgical interventions for CSF dynamics disorders, emphasizing the transition from established methodologies to innovative approaches. Current non-surgical treatment strategies primarily encompass three directions: pharmacological therapy, physical therapy, and biological regulation therapy. In terms of pharmacological interventions, developments from traditional diuretics to novel small-molecule drugs show promising therapeutic potential. In physical therapy, innovative techniques such as lower body negative pressure, transcranial magnetic stimulation, and vagus nerve stimulation have provided new options for clinical practice. Meanwhile, biological regulation therapy, exemplified by recombinant VEGF-C administration, has established novel therapeutic paradigms. These therapeutic strategies have demonstrated potential in improving CSF dynamics and enhancing CSF waste elimination. Future research should focus on developing individualized treatment protocols, elucidating of therapeutic mechanisms, and assessing longitudinal outcomes. This will facilitate the development of more precise therapeutic strategies and exploration of optimized multimodal treatment combinations in handling the so-called convert CSF dynamics dysfunction.

## 1 Introduction

An increasing body of research demonstrates that cerebrospinal fluid (CSF) dynamics dysfunction frequently coexists with various neurological diseases and often constitute an integral component of their pathophysiological mechanisms. Kimihira et al. (1) observed that asymptomatic ventriculomegaly with features of idiopathic NPH on MR imaging (AVIM) is not uncommon among elderly individuals, and long-term follow-up studies revealed that 62.5% of these cases progressed to normal pressure hydrocephalus (NPH) (2). This finding suggests the prevalence of subclinical CSF dynamics disorders, which termed as “Pre-Normal pressure hydrocephalus (Pre-NPH) by Kimihira et al. (1), or what we refer to as covert CSF dynamics dysfunction, is substantial. The Japanese studies from another perspective, indicates that covert CSF dynamics dysfunction does indeed impair cognitive and motor functions, though subtly but progressively, regardless of the victim is on concurrent neurological conditions or not. In the elderly population, the probability of existing or previous vascular pathologies, degenerative diseases, and infectious conditions is significantly higher compared to younger demographics. Consequently, their glymphatic system and CSF drainage mechanisms sustain cumulative damage through successive disease processes. Additionally, age-related deterioration leads to decreased operational efficiency, substantially increasing the likelihood of CSF dynamics disorders. Given the accelerating global demographic aging, the prevalence of covert CSF dynamics dysfunction is expected to rise, warranting increased attention.

Recent years have witnessed breakthrough discoveries in CSF circulation system research. Notably, the identification of the brain’s glymphatic system in 2012 fundamentally changed our understanding of CSF dynamics (3). This review examines the development of subclinical CSF dynamics dysfunction, associated pathologies, detection methodologies, and therapeutic advances, with particular emphasis on novel treatment strategies including physical therapy and biological regulation therapy.

## 2 Overview of CSF circulation: from traditional concepts to contemporary perspectives

CSF serves multiple essential functions in the central nervous system (CNS), including mechanical cushioning, mechanical protection, intracranial pressure regulation, metabolic homeostasis maintenance, and nutrient delivery.

The classical understanding of CSF dynamics portrayed a unidirectional system characterized by three primary processes: secretion, circulation, and absorption. This traditional model proposed that CSF, primarily produced by the choroid plexus (CP), follows a defined anatomical pathway through the ventricular system, i.e., from lateral ventricles to the third ventricle, via the Sylvian aqueduct to the fourth ventricle, ultimately exiting through the Magendie and Luschka foramina (4).

This circulation was traditionally thought to be regulated by pressure gradients, particularly between arachnoid granulations (AGs) and venous sinus (5). Following Starling’s law of filtration, hydrostatic pressure differentials among blood, CP epithelium, and ventricular spaces were considered the primary driving forces (5). Pressure-dependent adaptations include the expansion of arachnoid villi surface area during increased CSF pressure (6) and enhanced CSF secretion during pressure reduction (7).

Contemporary research has fundamentally transformed this understanding, revealing a more sophisticated model of CSF dynamics. The glymphatic system demonstrates size-dependent functionality (determined by astrocytic tight junctions), unidirectional flow (arterial to venous perivascular spaces), and spatiotemporal regulation (exhibiting regional specificity and circadian rhythm dependence) (8, 9). This revised model emphasizes the critical role of CSF-ISF exchange in maintaining CNS homeostasis and introduces meningeal lymphatic drainage as a principal clearance pathway, challenging traditional concepts of CSF circulation.

### 2.1 CSF production

CSF production represents a complex, multifactorial physiological process. While the CP composed of specialized vascularized epithelial cells within the ventricular system, has traditionally been considered the primary source of CSF production, emerging evidence increasingly recognizes interstitial fluid (ISF) derived from brain parenchyma as a significant contributor to CSF formation (10). Using uDISCO tissue clearing technique, and imaging with light-sheet, confocal and bright field light microscopy, recently studies have also found out that key proteins for choroidal CSF production, i.e., aquaporin 1 (AQP1) and Na+/K+/2Cl- cotransporter 1 (NKCC1), are widely or specifically distributed in the leptomeningeal vasculature of the intact brain, and lead to the increasingly recognized extra-choroidal CSF production (11, 12) The CSF secretory process is orchestrated by diverse regulatory mechanisms, predominantly physical factors including arterial pulsation, respiratory dynamics, postural alterations, jugular venous pressure fluctuations, and physical activity (10), as well as functional hyperemia and dietary influences (13, 14).

From a neurological regulatory perspective, the autonomic nervous system exerts precise control over CSF secretion through antagonistic mechanisms: sympathetic activation suppresses secretion, while cholinergic stimulation enhances it (9). The CP epithelial surface exhibits a diverse array of receptor populations, including those for dopamine, serotonin, melatonin, atrial natriuretic peptide, and arginine vasopressin, establishing a molecular framework for neurotransmitter and neuropeptide-mediated regulation of CSF secretion. And at the molecular level, mitochondria-enriched endothelial cells comprising the blood–brain barrier (BBB) express a sophisticated array of ionic and molecular transport systems (15). These transport mechanisms, encompassing cytoplasmic carbonic anhydrases, aquaporins (notably AQP1) (7), and membrane carrier proteins, collectively contribute to approximately half of CSF production through coordinated water transport and vectorial ion movement (Na+, Cl-, HCO3-, and K+) (16). Notably, direct inhibition of the Na+/K + ATPase transporter at the apical surface can diminish CSF secretion by up to 80% (17), underscoring how the expression and functional status of these transporters directly modulate CSF production.

### 2.2 Glymphatic CSF transport system

Recent advances in multifocal multiphoton microscopy and *in vivo* imaging technologies have revolutionized our understanding of glymphatic and CSF drainage system architecture. Fluorescent tracer studies have revealed an intricate fluid clearance network, comprising the glymphatic system, meningeal lymphatic pathways, and paravascular routes along cerebral arterial basement membranes (intramural peri-arterial drainage- IPAD pathway) (18). As a result, the theory that CSF mainly flows through the ventricular system and subarachnoid space and that CSF mainly re-absorbed via AGs has been greatly enriched. The vast extracellular and perivascular spaces have been incorporated into the CSF circulation system.

The glymphatic and CSF drainage system’s distinctive characteristic lies in its specialized fluid exchange mechanism: CSF enters the brain parenchyma via perivascular spaces (PVS) and facilitated by polarized aquaporin-4 (AQP4) channels on astrocytic endfeet, traverses the interstitial space between arterioles and venules, mediating CSF-ISF exchange while eliminating both soluble and insoluble waste products and macromolecules (19). This process encompasses periarterial CSF influx, interstitial solute movement, and interstitial solute drainage. Contemporary theory suggests that CSF transport occurs through bulk flow in the PVS, driven by cardiac cycle-dependent peristaltic pumping (20). This mechanism is partially dependent on brain tissue rigidity and permeability, with increased intracranial arterial pulsatility correlating positively with enhanced CSF flow volume. Alternative mechanisms include cardiac cycle-induced variations in arterial and vascular diameter (21). Additionally, glymphatic transport exhibits regional heterogeneity and postural dependence (22), with emerging evidence suggesting AQP4-dependent convective forces in CSF circulation (23). Collectively, these mechanisms facilitate CSF influx along periarterial pathways, intraparenchymal PVS, and white matter tracts. AQP4 polarization enables directional CSF flow, facilitating interstitial solute exchange and metabolite clearance via subarachnoid spaces and cisternal compartments. Ultimate CSF solute clearance occurs predominantly through meningeal lymphatic vessels (MLVs), rather than the traditionally proposed AGs and venous sinuses (24).

These revolutionized discoveries, urges researchers to re-examine the role of CSF circulation.

#### 2.2.1 Critical role of AQP4 in glymphatic transport dynamics

AQP4, a specialized water channel protein, is fundamental to glymphatic system function. Its distinctive feature is the capacity for bidirectional transcellular water transport in response to passive osmotic gradients. AQP4 exhibits highly polarized distribution at astrocytic endfeet-fluid interface boundaries, with significantly enhanced expression on luminal surfaces compared to non-endfeet membranes (15).

This specialized distribution pattern optimizes CSF movement between periarteriolar spaces (PAS) and PVS, facilitating waste elimination and molecular exchange. AQP4-knockout studies have demonstrated markedly reduced CSF water diffusivity and impaired cortical tracer clearance (25). AQP4 deletion, whether genetic or chemical, disrupts glymphatic CSF-ISF exchange and impairs macromolecular clearance, including amyloid-beta (Aβ) and tau proteins. Studies suggest that astrocytic AQP4 deficiency may reduce glymphatic system clearance efficiency by approximately 70% (3).

Notably, local accumulation of waste products, such as tau protein following repetitive head trauma (26), reciprocally suppresses AQP4 expression and function (27). Furthermore, CSF circulation obstruction has been linked to metabolic disturbances, like the folate metabolism disruption (28). These findings emphasize AQP4’s crucial role in CSF dynamics, cellular metabolism, molecular exchange, and waste elimination (23).

#### 2.2.2 Primary functions of the glymphatic system: CSF-ISF exchange and waste elimination

The brain’s intensive metabolic activity generates substantial waste products, including lactate, ATP metabolites, adenosine, inorganic salts, and potentially neurotoxic macromolecules such as Aβ and tau proteins. Given neuronal sensitivity to environmental perturbations, efficient elimination of these metabolites is crucial for maintaining brain homeostasis and preventing neurotoxicity. The glymphatic system establishes an efficient clearance pathway, facilitating metabolic waste transport into the PVS and subsequent elimination via meningeal and cranial lymphatic routes. Studies of compromised glymphatic function in conditions such as microinfarcts and vascular dementia (29, 30) demonstrate significant PVS enlargement and abnormal protein accumulation, such as Aβ (31).

The glymphatic system exhibits distinct circadian rhythmicity, with peak function during sleep (32), corresponding to AQP4 activity patterns (33). Sleep maintains brain slow vasomotion, a critical CSF flow driver, with single-night sleep deprivation potentially elevating CSF metabolic waste levels (34–36), underscoring the system’s crucial role in waste clearance.

Furthermore, metabolic product accumulation compromises glymphatic system function, creating a detrimental feedback loop. That is, CSF circulation disruption not just alters local flow dynamics but significantly impacts the essential processes of CSF-ISF exchange and waste elimination. Consequently, disrupted CSF-ISF exchange is increasingly recognized as a fundamental pathological mechanism in various neurological disorders.

### 2.3 Meningeal lymphatic pathways and CSF drainage

The landmark discovery of dural lymphatic vessels by Louveau et al. (37) has fundamentally transformed our understanding of CSF drainage mechanisms. This finding extends beyond the classical concept of arachnoid granulations and villi, revealing that CSF egress occurs through multiple transdural routes, encompassing meningeal and nasal lymphatics, as well as pathways along the dural sheaths of cranial and spinal nerves.

Comprehensive investigations have elucidated three principal CSF lymphatic drainage pathways: (1) the paravascular pathway, where CSF traverses periarterial, periarteriolar, and pericapillary spaces before entering either the venous circulation or deep cervical lymph nodes (dCLNs) via venous vessel walls; (2) the intramural periarterial drainage (IPAD) pathway, which utilizes the basement membranes within capillaries and arterial tunica media to access dCLNs; and (3) a direct transcytotic pathway across the blood–brain barrier (BBB) (38). These drainage routes have been extensively validated through multiple experimental paradigms, including selective lymphatic vessel ligation to dCLNs and genetic models with ablated dural lymphatic vasculature (39–41).

Significantly, impairment of meningeal lymphatic function not only inhibits CSF tracer penetration into PVS and brain parenchyma but also compromises parenchymal tracer clearance (42). This bidirectional impact underscores the critical importance of maintaining optimal functionality in the intricate meningeal and skull base lymphatic drainage network.

### 2.4 Vulnerabilities of the CSF circulation

The glymphatic system and CSF lymphatic pathways primarily comprise PVS, basement membrane compartments, and nerve sheaths – intricate microchannels that remained undetected by conventional microscopic techniques. While AQP4 enables astrocytic networks to encompass blood vessels and establish CSF pathways interconnecting regional PVS, these passive fluid channels exhibit high dependency on osmotic gradients (43).

This sophisticated architecture demonstrates particular vulnerability to pathological conditions. The ultrastructural gaps (< 20 nm) between astrocytic end-feet serve a critical filtration function while simultaneously imposing substantial resistance to fluid and solute transport (18). This architectural characteristic facilitates relatively unrestricted movement of smaller molecules (e.g., Texas Red Dextran) through the interstitial fluid space, while larger molecules (such as 2000 kDa FITC-dextran) are restricted to rapid transit through subarachnoid and periarterial channels without ISF penetration (44). In pathological states where cellular debris, amyloid-*β*, and tau proteins accumulate, the brain’s inherently limited clearance capacity becomes further compromised. And the morphological integrity of PVS, determined by astrocytic-vascular interactions, becomes compromised during vascular alterations and astrocytic edema, consequently impacting glymphatic system functionality (16).

The system’s driving mechanisms exhibit inherent fragility. Cardiac pulsations demonstrate greater amplitude near proximal major vessels, while respiratory fluctuations predominate in the brainstem and demonstrate widespread cortical distribution (45). The complex interplay of arterial wall expansion, rigid motions, and static CSF pressure gradients generates both net and oscillatory PVS flow patterns (46). This intricate flow dynamics becomes readily disrupted in disease states, subsequently affecting metabolic waste clearance efficiency. While the recently characterized arachnoid cuff exit (ACE) points, where bridging veins traverse the arachnoid barrier, facilitate molecular exchange (47), their anatomical accessibility paradoxically increases the system’s susceptibility to pathological perturbations.

**Figure 1:**
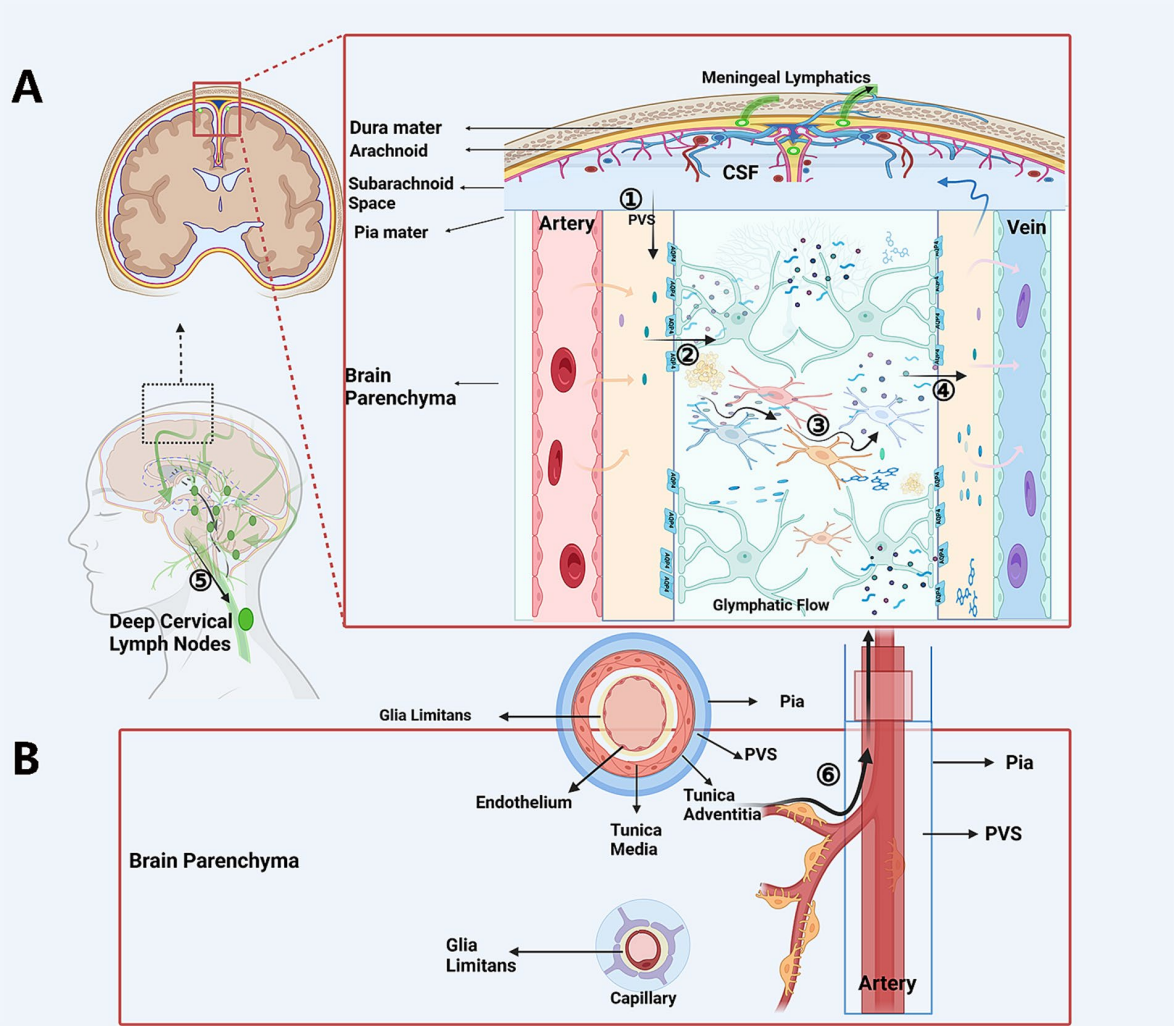
The schematic CSF circulation and possible sites of “flow-jam”. In glymphatic scenario **(A)** CP-produced and extra-CP-produced CSF is driven into the periarterial space **(A)**: CSF trans-passes from the periarterial spaces into interstitial tissue, transports through the “forest” of neurons and glias via extracellular spaces, exchanges with ISF, and carries soluble or non-soluble waste and penetrate into the perivenous spaces. The CSF is the then drained via the lymph vessels in dura matter, skull base, and via arachnoid granules into the sinus, and then further to the dCLNs. In the IPAD pathway scenario **(B)**. ISF with soluble or non-soluble waste passes out of the brain along basement membranes in the walls of capillaries, along basement membranes surrounding smooth muscle cells in the tunica media of arterioles and arteries, and then to the surface of the brain, and is finally drained out through perivascular lympatic drainage to the dCLNS. Possible jam sites and common reasons: ① Periarterial spaces, because the jam of non-soluble waste, the decreased driving force, and collapse or destruction of this structure. ② At the astrocyte endfeet-pia mater surface on the periarterial space side, because of decreased expression of AQP4 and AQP4 depolarization. ③ Deformed geometries and increased tortuosity and increase sticky of the ISF because of the accumulation of waste in the inter-cellular space. ④ At the astrocyte endfeet and perivenous spaces luminal surface interface, due to the decreased expression of AQP4 or AQP4 depolarization on the astrocyte endfeet. ⑤ The lymph vessels for CSF drainage, for being obstructed by tangible materials or compromised by inflammation or degenerative procedures secondary to aging or diseases. CP, choroid plexus; CSF, cerebrospinal fluid; dCLNs, deep cervical lymph nodes.

**Figure 2:**
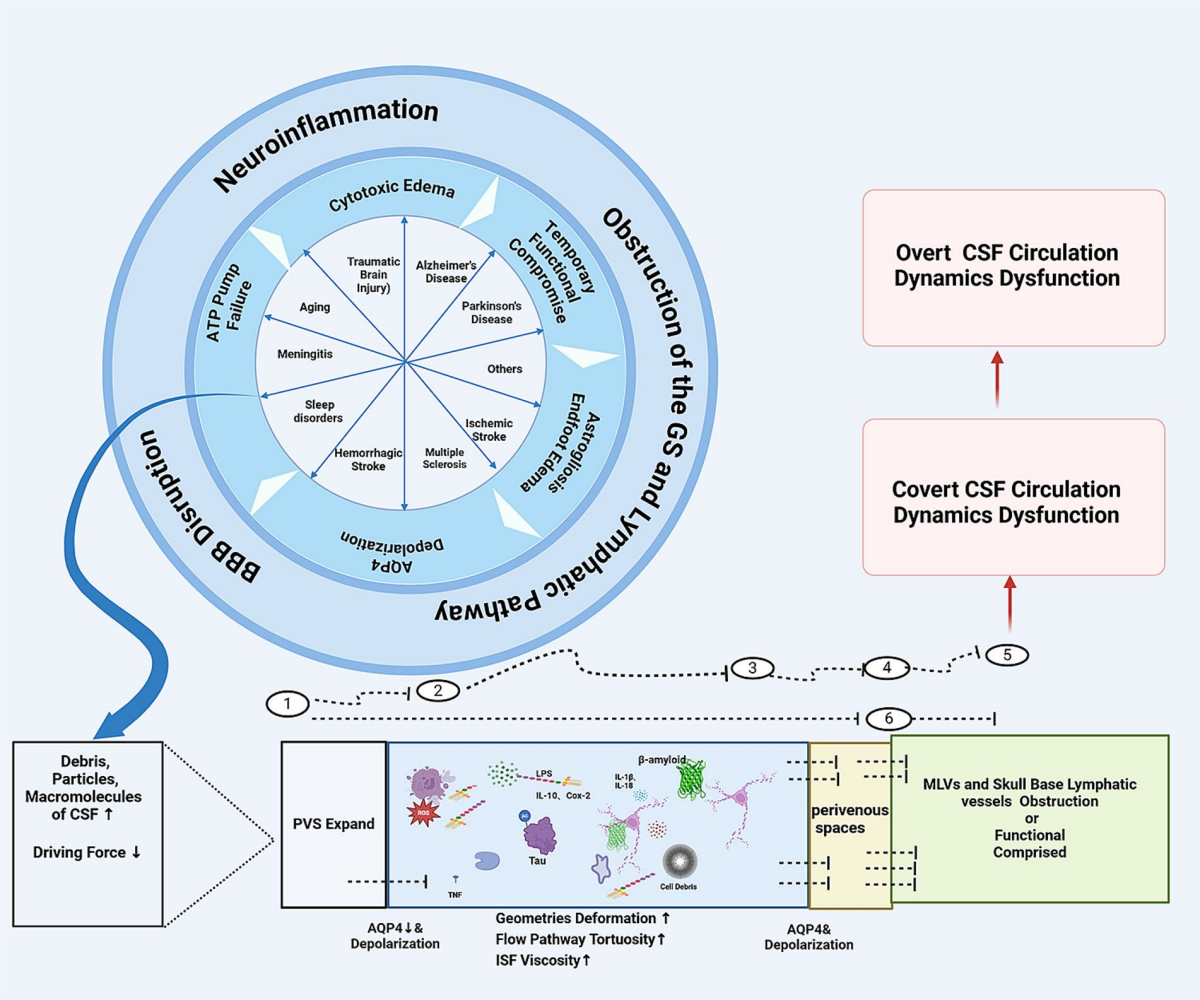
The schematic diagram of how the CNS diseases might jam the glymaphatic system and IPAD pathway and cause CSF circulation dynamics dysfunction: ① Periarterial spaces. ② At the astrocyte endfeet-pia mater surface on the periarterial space side, ③ Within the interstitial tissues. ④ At the astrocyte endfeet and perivenous spaces luminal surface interface. ⑤ Meningeal lymphatic vessels or skull base lymphatic vessels. ⑥ IPAD pathway. CSF, cerebrospinal fluid; PVS, perivascular spaces; MLVs, meningeal lymphatic vessles.

In sum, the CSF actually operates through a system consisted of the glymphatic system, the IPAD pathway, lymphatic vessels in the dura mater and the skull base. The above-mentioned glymphatic and lymphatic drainage system are slender and highly dependent on the amount and polarization of aquaporins (e.g., AQP1, AQP4) and the expression of water transport molecular such as the NKCC1 molecules, as well as on driving force of the cardiac and arterial pulsation and respiratory movement, to facilitate the CSF transportation, CSF-ISF exchange, and CSF-mediated waste removal. The structure of the glymphatic and lymphatic drainage system is both ingeniously designed, endow the system to provide energy nourishment and functional refreshing for the dense, metabolically active, functionally complex CNS tissues that are encaved in limited space, while coordinating the circadian clock in a delicate manner. However, this system is also prone to being clogged by mechanical blockages of debris, particles and macromolecules, and being destructed and/or functionally compromised by the sub-sequential neuroinflammation, oxidative stress and depolarization caused under the combined effects of aging or diseases, leading to covert or overt CSF circulation dysfunction (Figures 1, 2).

## 3 Neurological disorders associated with CSF dynamic dysfunction

Accumulating evidence demonstrates a robust association between CSF circulation impairment and diverse neurological conditions. Disease-induced structural and functional alterations in perivascular and intraarterial infrastructure compromise glymphatic system waste clearance efficiency, precipitating a cascade of pathological events including cerebral edema, secondary inflammation, neurodegeneration, and further systemic imbalance (28).

Conversely, compromised clearance of brain metabolic waste, whether due to aging, cerebrovascular insufficiency, or other pathological factors, can significantly influence disease onset and progression (48). Cerebral amyloid angiopathy serves as a paradigmatic example of this bidirectional relationship (49). Notably, studies indicate that glymphatic dysfunction may constitute a prerequisite condition for dementia development in neurodegenerative disorders such as Alzheimer’s Disease (AD) and Parkinson’s disease (PD) (29, 30). This hypothesis has been further substantiated through animal models of traumatic brain injury (50, 51) and multiple microinfarcts (29, 30), which demonstrate significant glymphatic system impairment. It is now increasingly convinced that CSF dynamic dysfunction represents a common pathophysiological mechanism underlying numerous neurological conditions.

### 3.1 Age-related alterations in CSF dynamics

Studies have demonstrated reduced CSF production and pressure in aged mice, with diminished viral vector clearance indicating global compromise of glymphatic system function during aging (52). Aging emerges as a predominant modulator of glymphatic system functionality, with its effects manifesting across multiple mechanistic levels.

Primary age-related alterations occur in the molecular architecture of the system. Most notably, aging is associated with decreased AQP4 polarization in astroglial endfeet surrounding cortical penetrating arterioles (53), primarily due to disrupted astrocyte-pericyte interactions. Secondarily, age-associated modifications in cerebral microvasculature, including basement membrane thickening and BBB deterioration, result in PVS dysfunction or enlargement, potentially facilitating waste accumulation (15). While aging may not directly alter cerebral arterial diameter, it significantly attenuates pulsatile blood flow due to increased arterial wall rigidity (54). These alterations in arterial pulsatility and AQP4 polarization may explain the observed mismatch between glymphatic influx and efflux dynamics.

Tertiary effects manifest in the impairment of MLV integrity, compromising CSF drainage from meningeal lymphatics to dCLNs (55). The structural integrity, density and function of meningeal lymphatics regress during aging (56). Furthermore, research has established a correlation between pial thickness and enhanced CSF flow, with reduced Aβ deposits in the PVS of aged mice (57). Age-related pial atrophy and the emergence of pial denudation regions, absent in young animals (57), contribute to system dysfunction.

From a systems dynamics perspective, age-associated vascular conditions, including hypertension, arteriosclerosis, and vascular remodeling, collectively diminish arterial pulsatility (20). Hypertension, specifically, increases backflow while reducing net perivascular space flow, consequently diminishing CSF production, pressure, and CSF-ISF exchange efficiency (20). This hypertensive impact on glymphatic system function manifests in both early and advanced disease stages. The glymphatic system dysfunction precipitated by various age-related pathophysiological processes ultimately compromises CSF waste clearance capacity, potentially initiating a detrimental cycle of disease progression (58).

### 3.2 Cerebrovascular diseases

Cerebral infarction, characterized by sudden interruption of blood supply, results in ischemic necrosis of brain tissue. The high lipid content of brain tissue, particularly myelin, presents significant challenges for proteolytic degradation. While neutrophils recruited post-infarction are rapidly cleared by activated microglia (59), this process actually reduces overall cellular debris clearance efficiency (60). Additionally, cellular debris clearance is further impeded by insufficient “find me” and “eat me” signals, and the requirement for cholesterol transporters in myelin debris degradation (61, 62). Recent observations by Zhou et al. (63) revealed that microvascular endothelial cells, typically not involved in phagocytosis, participate in necrotic material clearance following CNS injury, suggesting that post-injury debris accumulation usually exceeds phagocytic capacity. Zbesko et al. (64) demonstrated that clearance of liquefactive necrotic brain tissue post-infarction requires at least seven weeks and depends on microglial endocytosis within PVS. Furthermore, due to complex neural connectivity, cerebral infarction induces secondary remote degeneration (65), prolonging macromolecules-granular material generation.

The efficiency of CSF drainage and clearance of particulate matter, debris, and metabolic products is crucial for brain tissue health. Research has shown that cholesterol crystal-induced multiple microinfarcts can cause abnormal CSF tracer transport through the glymphatic system persisting beyond 14 days (30), leading to significant PVS expansion and cognitive decline (29). Recent studies by Howe et al. (66) confirmed reduced post-stroke CSF influx through the glymphatic system, indicating system dysfunction. Egorova et al. (67) observed progressive lateral ventricle enlargement in ischemic stroke patients. Lymphatic obstruction post-infarction may lead to asymptomatic ventriculomegaly with features of idiopathic normal pressure hydrocephalus on MR imaging (AVIM) or pre-normal pressure hydrocephalus (Pre-NPH), as reported by Kimihira et al. (1), potentially causing cognitive and motor impairments.

In hemorrhagic strokes, particularly subarachnoid hemorrhage, substantial erythrocyte accumulation in cervical lymph nodes and meningeal lymphatics temporarily disrupt lymphatic clearance. This disruption exacerbates neuroinflammation, neuronal death, elevated CSF pressure, neurological and cognitive deficits, and potential hydrocephalus development (68, 69). In addition, chronic cerebral hypoperfusion associated with small vessel disease increases BBB and blood-CSF barrier permeability to blood-borne macromolecules, triggering neuroinflammation (70, 71) and reducing AQP4 polarity (72), thereby disturbing glymphatic transport. Similar pathological patterns are observed in vascular dementia patients (73).

Taken together, cerebrovascular diseases significantly impact CSF dynamics through multiple interconnected pathophysiological mechanisms. Primary vascular risk factors, including hypertension and arterial stiffness, lead to reduced cardiac pulsatility and subsequent impairment of CSF flow dynamics. These changes are particularly evident in patients with arteriosclerosis and stenosis of intra- and extra-cranial arteries. Furthermore, acute vascular events trigger a cascade of pathological processes, including glymphatic system disruption, accumulation of cellular debris and macromolecules, and neuroinflammatory responses. This complex interplay creates a self-perpetuating cycle where CSF dynamic dysfunction becomes both a consequence of cerebrovascular pathology and a contributing factor to disease progression, establishing itself as a critical mediator in the pathogenesis of cerebrovascular disorders.

### 3.3 Traumatic brain injury (TBI)

Accumulating evidence demonstrates post-TBI glymphatic system flow disruption, partially attributed to AQP-4 channel redistribution away from astrocytic endfeet, compromising waste removal capacity (50, 51). Even mild TBI can disrupt the correlation ship lies between ventricular clearance of radiolabeled tracer and the ventricular blood flow (74). And multiple TBI-induced glymphatic dysfunction can persist chronically (26), with cumulative effects leading to neurodegeneration, cognitive decline, and behavioral changes, manifesting as chronic traumatic encephalopathy (75). Traumatic brain injury initiates a complex cascade of events that significantly impairs CSF dynamics, creating a bidirectional pathophysiological relationship. The acute mechanical impact disrupts the BBB and glymphatic system architecture, while secondary injury mechanisms trigger the accumulation of cellular debris, inflammatory mediators, and blood products within the CSF compartments. This accumulation, coupled with post-traumatic alterations in AQP4 expression and perivascular pathway dysfunction, compromises CSF circulation and waste clearance efficiency. The resultant CSF dynamic dysfunction not only exacerbates the initial trauma but also contributes to the expansion of secondary injury zones and chronic post-traumatic sequelae, highlighting the critical need for targeted therapeutic interventions to restore CSF homeostasis in TBI management.

### 3.4 Neurodegenerative disorders

Dysfunction of MLVs and the glymphatic system extends beyond aging, cerebrovascular disease, and traumatic brain injury, manifesting in various neurodegenerative conditions, notably AD (characterized by Aβ and tau protein accumulation) and PD (marked by *α*-synuclein aggregation) (76). Given that pathological protein accumulation represents a central pathogenic mechanism in neurodegenerative disorders, the glymphatic system and brain lymphatic drainage networks have emerged as crucial areas of investigation since their discovery (77).

Research has substantiated that compromised glymphatic function directly contributes to Aβ accumulation (32) and other neurotoxic protein aggregation (78). In experimental models utilizing intranigral injection of recombinant human α-synuclein or transgenic mice overexpressing human A53T-α-synuclein, glymphatic suppression through either AQP4 gene deletion or acetazolamide administration resulted in reduced clearance of injected α-synuclein from brain tissue or accelerated α-synuclein accumulation. Notably, A53T-α-synuclein overexpression was found to diminish AQP4 expression/polarization and suppress glymphatic activity (79). Furthermore, reduced glymphatic clearance has been documented to precede amyloid plaque formation in rodent models of AD (80).

These findings indicate that CSF circulation dysfunction is not merely a transient manifestation at specific disease stages in neurodegenerative conditions such as AD and PD. Rather, it represents an integral component throughout disease initiation and progression, warranting significant attention and targeted therapeutic intervention strategies.

### 3.5 Intracranial infections

Intracranial infections introduce not only pathogenic organisms into CSF compartments but also trigger a cascade of pathophysiological changes, including cytokine release, immune cell infiltration and proliferation, and various substances penetrating through compromised BBB or blood-CSF barrier interfaces. Studies in mice infected with neurotropic viruses have revealed that infection promotes MLV expansion while simultaneously impairing MLV-mediated macromolecular drainage and viral clearance from the CNS to dCLNs. Similar dysfunction patterns have been observed in intracranial bacterial infections (81).

In experimental models using Wistar rats with pneumococcal meningitis, the retention of Evans blue-albumin (EBA) within CSF compartments indicated significant glymphatic system impairment. This dysfunction likely results from the detachment of astrocytic end-feet from BBB vascular endothelium, leading to AQP4 mis-localization. The resultant compromised solute drainage and accumulation of neurotoxic bacterial components within cerebral CSF compartments ultimately precipitate widespread neuroinflammation and neuronal injury (82).

Complementary research by Li et al. (52) demonstrated that disruption of CSF drainage, whether through surgical ligation of lymphatic vessels or photodynamic ablation of dorsal MLVs, exacerbated neurological damage and mortality in virus-infected mice. Conversely, prophylactic administration of vascular endothelial growth factor C (VEGF-C) attenuated viral infection-induced pathology. Collectively, these findings underscore the critical role of CSF circulation dysfunction in the pathogenesis and therapeutic management of intracranial infections.

### 3.6 Two extreme settings of CSF dynamic dysfunction and the underlying inspiration

#### 3.6.1 Idiopathic normal pressure hydrocephalus (iNPH)

iNPH represents a complex neurological disorder intrinsically linked to CSF circulation dysfunction (83). Comparative analyses of intracranial pressure (ICP), CSF outflow resistance (Rout), and cardiac-related ICP pulsation amplitudes (AMPs) between iNPH patients and health controls have revealed that while ICP values remain comparable, a subset of iNPH patients demonstrates significantly elevated Rout. Additionally, at resting pressure, iNPH patients exhibit a trend toward increased AMPs.

Impaired CSF influx and efflux patterns have been documented in iNPH patients (84), accompanied by accumulation of brain metabolic waste products and Aβ plaques, perivascular reactive astrogliosis, and aberrant localization of astrocytic AQP4 (85, 86). These findings suggest that altered CSF dynamics may initiate a self-perpetuating pathogenic cycle in iNPH development. Although CSF turnover rates reportedly remain unchanged in iNPH patients (87), excess CSF accumulation in dilated PVS induces compression of parenchymal penetrating arteries, resulting in diminished pulsatility (88). Furthermore, dysregulation of aquaporin expression, characterized by elevated AQP-1 and reduced AQP-4 levels, contributes to BBB dysfunction in iNPH patients (89–91). These pathophysiological mechanisms likely operate synergistically, cutting down fluid speed in the arterial perivascular space and the amount of the CSF pass through the glymphatic pathway, raising in resistance as measured with infusion tests (88) and therefore compromising waste clearance and promoting toxic metabolite accumulation (92),

Sleep disturbances are frequently observed in iNPH patients, representing a significant clinical manifestation (86). Given that sleep plays a crucial role in glymphatic system function (93), these sleep abnormalities may constitute an important pathophysiological mechanism in iNPH progression through impaired CSF circulation.

The pathophysiology of iNPH reveals a complex interplay between glymphatic transport and CSF outflow mechanisms. This condition is characterized by concurrent alterations in both CSF inflow/outflow dynamics and lymphatic drainage, creating a self-perpetuating cycle of dysfunction. The bidirectional relationship between sleep quality and glymphatic function further complicates this picture: sleep disruption impairs glymphatic clearance, while compromised CSF dynamics may adversely affect sleep architecture. This intricate relationship exemplifies how subtle alterations in CSF dynamics can significantly influence the progression of neurological disorders. Simultaneously, it has been noticed that patients with iNPH achieved real-time cognitive improvement on ultra-fast neuropsychological testing during infusion and tap test (94), which further support the significance of dealing with covert CSF disorders that accompany central nervous system diseases.

#### 3.6.2 Idiopathic intracranial hypertension (IIH)

Wardman et al. (14) demonstrated in experimental models that female rats subjected to either a 21-week high-fat diet (HFD) or 28-day adjuvant testosterone treatment exhibited distinct pathophysiological patterns. HFD-fed rats displayed elevated ICP and increased Rout by 50%, without alterations in CSF production. Conversely, chronic testosterone administration induced elevated ICP, enhanced CSF secretion rate (85%), and increased Na+, K+, 2Cl- cotransporter (NKCC1) activity in CP. Both interventions successfully replicated clinical IIH features, suggesting that impaired CSF drainage capacity and augmented CSF production represent parallel pathogenic mechanisms in IIH, intrinsically linked to glymphatic system dysfunction.

Molecular investigations by Alimajstorovic et al. (95) revealed that inflammatory mediators (CCL2, TNF-*α*), hormonal factors, and dietary components modulate both CSF secretion and drainage resistance. These findings align with documented structural alterations, including astrogliosis, altered AQP4 expression patterns, and compromised BBB integrity (96).

Advanced neuroimaging and CSF tracer studies have demonstrated impaired perivascular and ISF dynamics in IIH patients. This dysfunction manifests as delayed tracer clearance and increased prevalence of enlarged perivascular spaces (EPVS) (167), indicating compromised glymphatic function.

The pathogenesis of IIH represents a complex interplay between HFD-induced obesity, cytokine dysregulation, metabolic/hormonal disorders, and adipose tissue dysfunction (97), collectively compromising glymphatic system function. Moreover, structural anomalies such as cerebral venous sinus and internal jugular vein stenosis impair AG and glymphatic system function (98). Conversely, elevated ICP induces extramural venous sinus stenosis (99) and reduces cerebrovascular pulsatility (100), establishing a self-perpetuating cycle of glymphatic dysfunction, elevated ICP, and progressive system impairment.

That is, CSF circulation may seem like isolated circulation, but in fact, systemic factors such as metabolism and hormones have a significant impact on it. Once CSF dynamic dysfunction is established, it’s impact on the system is not limited within the intracranial cavity. These findings underscore the intricate relationship between systemic metabolic homeostasis and CSF dynamics, highlighting the need for comprehensive therapeutic approaches that address both local and systemic pathophysiological mechanisms in CSF dynamics management.

In summary. Different central nervous system diseases, although their pathophysiological mechanisms are all different, there might be something that is the same among them. That is, regardless of whether the damaging stimulus is acute or slow, whether it leads to acute necrosis or slow degeneration, whether it causes a large amount of acute cellular debris and toxic products to be produced in a short term or whether these substances are produced in small amounts but continuously, the debris, particles, macromolecules might all are capable of imposing a transient or persistent overload stress on the glymphatic system/lymphatic drainage system of CSF, causing the latter to be blocked at the points of jam described in Figure 1, triggering secondary inflammation, degeneration and aging processes, forming covert CSF circulation disorders (Figure 2). If the impaired glymphatic/lmphatic system are unable to fix the above overload, or does not receive timely treatment intervention, it then enters a vicious cycle and develop into overt CSF circulation disorders, for example, normal pressure hydrocephalus.

## 4 Detection of covert CSF dynamic dysfunction

Prior to the development of normal pressure hydrocephalus or even ventricular enlargement, as described by Iseki et al. (2) and Kimihira et al. (1), covert CSF circulation abnormalities are subtle and mild. PVS integral components of the glymphatic system, participate in maintaining ISF circulation. While normal PVS are typically undetectable on MRI, EPVS become visible, enabling indirect assessment of lymphatic system function through imaging (16).

In the context of covert CSF circulation dysfunction, MRI-detected dilated PVS serve as biomarkers for glymphatic dysfunction and waste accumulation (101). Shao et al. (102) and Taoka et al. (103) introduced a non-invasive methodology in 2017 – diffusion tensor imaging along the PVS (DTI-ALPS) – for evaluating glymphatic system function in human brain, which has gained widespread acceptance. Subsequent research has refined ALPS calculation methods (mALPS), demonstrating comparable efficacy to DTI-ALPS in representing glymphatic system function (19), though further validation of mALPS correlation with human glymphatic system function remains necessary.

Albayram et al. (104) developed a three-dimensional fluid-attenuated inversion recovery (FLAIR) MR method, providing detailed visualization of MLVs, CSF/ISF drainage patterns around neural structures, and dCLNs without requiring contrast agents. And phase-contrast MRI (PC-MRI) enables investigation of arterial peak flow, pulsatility index, and resistive index in arterial and venous compartments, while also measuring arteriovenous and CSF peak flow and stroke volume to assess cerebral hydrodynamics and hemodynamics (105).

CSF circulation abnormalities manifesting as widened subarachnoid space (SAS) can be detected through near-infrared transillumination/backscattering sounding (NIR-T/BSS) and MRI, with NIR-T/BSS capable of detecting CSF pulsatility changes in the SAS (106). Recent work by Rossinelli et al. (107) employed large-scale computational fluid dynamics (CFD) analysis to investigate CSF dynamics and geometrical alterations in the optic nerve subarachnoid space, utilizing supercomputing to analyze hydraulic resistance, volumetric rate, and microstructural changes.

Chen et al. (108) introduced CSF-based spatial statistics (CBSS), leveraging low b-value MRI to quantify CSF motion variance and enable automated quantification of CSF pseudo-diffusivity in individual sulci, cisterns, and ventricles. This technique facilitates auto-segmentation and quantitative spatial analysis of CSF pseudo-diffusion.

Contrast-enhanced MRI using specific CSF tracers (e.g., gadobutrol) enables visualization of glymphatic system entry and clearance pathways (93, 109, 110). The development of “glymphatic MRI (gMRI)” enables detailed evaluation of CSF circulation through sequential imaging before and after intrathecal tracer injection, allowing precise ventricle reflux grading and tracer clearance measurement (111, 112). However, the invasive nature of intrathecal tracer administration presents limitations to widespread application.

When lumbar puncture is indicated, semiautomatic CSF infusion studies can assess CSF formation rate (87). Additionally, CSF Lipocalin-type prostaglandin D synthase levels may serve as indicators of glymphatic system health (113).

In preclinical studies, Mee-Inta et al. (114) employed high-frequency ultrasound (HFUS) with DiI-loaded microbubbles and FePt@PLGA nanoparticle contrast agents to evaluate meningeal lymphatic system flow, enabling real-time statistical analysis of HFUS signals at dCLNs.

## 5 Non-surgical therapeutic strategies for CSF dynamic dysfunction

The therapeutic landscape for CSF dynamic dysfunction has evolved significantly, expanding beyond traditional surgical interventions to encompass diverse non-invasive approaches. Contemporary treatment strategies target three fundamental aspects: CSF secretion modulation, circulation enhancement, and drainage optimization. These approaches include innovative neuromodulation techniques, pharmacological interventions, and physical therapeutic modalities.

Neuromodulatory approaches, including vagus nerve stimulation (VNS), transcutaneous auricular VNS, and non-invasive brain stimulation techniques, demonstrate promising efficacy in regulating CSF dynamics. Physical interventions, such as lower body negative pressure (LBNP) and continuous positive airway pressure (CPAP), offer novel mechanisms for enhancing CSF circulation and clearance.

Pharmacological strategies have advanced from traditional carbonic anhydrase inhibitors to more targeted approaches, including selective membrane transport protein modulators and novel small molecule compounds. Recent developments in AQP-4 modulation and recombinant VEGF-C therapy represent significant progress in enhancing glymphatic function and meningeal lymphatic drainage.

These emerging non-surgical interventions are potentially offering more physiological approaches to CSF dynamic dysfunction management. The continued development of these strategies reflects a growing understanding of CSF dynamics and the complex interplay between various regulatory systems in maintaining CSF homeostasis.

### 5.1 Non-medication therapeutic strategies

#### 5.1.1 Low-fat diet

Dietary intervention represents a non-invasive therapeutic approach with significant potential for modulating CSF dynamics. Although evidence regarding dietary effects on CSF secretion and drainage resistance remains controversial (14, 95), clinical observations have demonstrated that CP volume and microstructural characteristics are influenced by obesity-induced inflammation, dietary factors, and related dysregulatory mechanisms (115). Furthermore, weight reduction, whether achieved through bariatric surgery or community-based weight management programs, has been shown to improve intracranial CSF circulation (116).

Current clinical guidelines recommend a multicomponent lifestyle intervention combining dietary modification, physical activity, and behavioral therapy as the primary approach for achieving modest weight loss in patients with BMI <35 kg/m2. For individuals exceeding this threshold, bariatric surgery warrants consideration (117).

The observed gender-specific variations in treatment response may be attributed to complex interactions between sex hormones and lipid metabolism (118). This relationship holds particular significance for women of reproductive age, where low-fat dietary interventions may exert dual benefits: weight management and modulation of CSF production and circulation through lipid metabolism regulation. This non-invasive therapeutic approach, characterized by its safety profile and implementation feasibility, can serve as a foundational component of comprehensive treatment strategies.

However, several critical knowledge gaps persist regarding the long-term efficacy and mechanistic underpinnings of dietary interventions. Specifically, the regulatory effects of low-fat diets on CSF circulation may demonstrate significant heterogeneity across different age demographics and pathological conditions, presenting unique challenges for developing personalized therapeutic protocols.

#### 5.1.2 Neuromodulation therapy

##### 5.1.2.1 Vagus nerve stimulation (VNS) and transcutaneous auricular vagus nerve stimulation (ta-VNS)

VNS, an FDA-approved neuromodulation therapy widely employed in treating epilepsy, depression, and cluster headaches, has recently demonstrated novel potential in CSF circulation regulation. Cheng et al. (119) conducted fluorescent tracer (TxRed-3kD) studies in murine models, revealing significantly enhanced CSF penetrance in subjects receiving clinical VNS paradigms (30 Hz, 10% duty cycle) compared to naïve control and sham groups.

Ta-VNS, a non-invasive neuromodulatory approach targeting the cutaneous branch of the vagus nerve, has demonstrated efficacy in attenuating post-subarachnoid hemorrhage inflammatory responses while restoring impaired glymphatic system function and CSF clearance (120–122). Notably, VNS may influence CSF dynamics through autonomic nervous system modulation, warranting further mechanistic investigation for treatment protocol optimization.

##### 5.1.2.2 Non-invasive brain stimulation (NIBS)

NIBS has emerged as a promising therapeutic modality for modulating brain activity through various energy stimulation paradigms (123). Repetitive transcranial magnetic stimulation (rTMS) employs rapidly alternating magnetic fields to regulate neuronal electrical activity (124). Research demonstrates that this modulation facilitates tau protein drainage from CSF to dCLNs via MLVs, significantly reducing Aβ deposition in 5xFAD mouse models (125, 126).

A novel NIBS approach combining focused ultrasound with intravenous microbubbles (FUS-MB) enhances CSF clearance rates in dCLNs while reducing reactive gliosis in hippocampal and piriform cortical regions (127). Importantly, human applications have demonstrated comparable efficacy and safety profiles (128).

##### 5.1.2.3 Other neuromodulatory approaches

Transcranial photobiomodulation (tPBM), delivering red or near-infrared light photons transcranially, demonstrates stimulative, regenerative, and neuroprotective effects. Nine-day tPBM treatment protocols significantly enhance cranial and extracranial lymphatic system function while reducing neurotoxic waste accumulation (129), suggesting therapeutic potential for CSF outflow disorders (130). Notably, dark phase tPBM administration in rodent models demonstrates enhanced efficacy, underscoring sleep’s crucial role in brain waste clearance.

Recent research by Murdock et al. employed chemogenetic manipulation combined with multisensory 40 Hz gamma stimulation in 5XFAD mouse models of AD, confirming enhanced CSF influx and ISF efflux in cortical regions. This effect was attributed to vasoactive intestinal peptide interneuron activation, facilitating glymphatic clearance through arterial pulsatility modulation (131).

#### 5.1.3 Lower body negative pressure (LBNP)

Lower body negative pressure (LBNP), an innovative therapeutic modality derived from aerospace medicine research, emerged from observations of neuro-ophthalmic manifestations in astronauts exposed to prolonged microgravity. These manifestations, including headaches, papilledema, and visual impairment, parallel symptoms associated with elevated intracranial pressure, prompting investigation into gravitational influences on CSF dynamics (132).

Research by Lonnie et al. (133) demonstrated that LBNP application simulates gravitational effects by creating a controlled negative pressure environment around the lower body, facilitating ventricular fluid drainage and achieving significant pressure reduction in both brain parenchymal and ventricular-septal compartments. Their findings established that 20 mmHg represents the optimal LBNP threshold for ICP reduction while maintaining adequate cerebral perfusion, exhibiting a characteristic non-linear dose–response relationship. This non-invasive therapeutic approach presents promising potential for managing pathological ICP elevation, particularly in TBI cases.

Significantly, acute LBNP application does not induce alterations in choroidal thickness (134). Treatment efficacy demonstrates strong positional dependence, emphasizing the critical importance of maintaining optimal patient positioning, ensuring unimpeded jugular venous return, and avoiding cervical flexion or compression for maximizing therapeutic outcomes.

#### 5.1.4 Continuous positive airway pressure (CPAP)

Respiratory dynamics exert significant influence on cerebral CSF flow patterns. CPAP, a non-invasive respiratory support modality originally developed for obstructive sleep apnea management, maintains airway patency through mask-delivered continuous positive airflow. Recent investigations have revealed CPAP’s multifaceted effects on CSF circulation beyond its primary role in sleep quality enhancement.

Studies demonstrate that CPAP modulates the dynamic equilibrium between jugular venous return and intracranial pressure through increased intrathoracic positive pressure. Clinical evidence indicates significant enhancement of glymphatic drainage system function in obstructive sleep apnea patients following one month of CPAP therapy (135). In preclinical studies, Ozturk et al. (136) demonstrated CPAP-induced augmentation of CSF flow velocity at the skull base and regional enhancement of glymphatic transport, establishing a theoretical framework for CPAP application in CSF circulation disorders.

Notably, therapeutic efficacy depends critically on individualized CPAP parameter optimization. Excessive airway pressure may compromise venous return, while suboptimal pressure fails to achieve therapeutic targets, necessitating careful pressure titration based on individual patient characteristics and systematic outcome assessment.

#### 5.1.5 Additional therapeutic approaches

Very low-intensity ultrasound (VLIUS), operating at 1 MHz center frequency with 1 kHz pulse repetition frequency, demonstrates significant enhancement of CSF tracer influx into PVS and facilitates interstitial macromolecular clearance from brain parenchyma without inducing tissue damage. Mechanistic studies suggest VLIUS enhances glymphatic influx through the transient receptor potential vanilloid-4-aquaporin-4 pathway in astrocytes (137).

Furthermore, hypothermia augments glymphatic influx through CSF flow rate modulation. Respiratory parameters (rate and amplitude) and cardiac function collectively contribute to glymphatic influx regulation (138).

### 5.2 Pharmacologic therapy

#### 5.2.1 Modulation of CSF secretion

Acetazolamide (AZA), a classical carbonic anhydrase inhibitor, maintains a pivotal position in CSF circulation disorder management. The European Headache Federation endorses it as first-line therapy for intracranial pressure reduction (139). AZA’s primary mechanism involves carbonic anhydrase inhibition within the CP, modulating HCO3- transport protein activity and consequently reducing CSF secretion (140).

However, AZA’s clinical application presents significant challenges. The ubiquitous expression of 15 carbonic anhydrase isoforms in mammalian systems (141) necessitates high-dose administration (4 g/day), potentially precipitating diverse adverse effects including sensory disturbances, gastrointestinal manifestations, polyuria, and depressive symptoms. Furthermore, its safety profile during pregnancy remains contentious, mandating careful consideration and collaborative decision-making between clinicians and patients (142, 143).

These therapeutic limitations have catalyzed research into more selective carbonic anhydrase inhibitors. Current investigations focus on identifying novel compounds with specific affinity for CP carbonic anhydrase, aiming to optimize therapeutic efficacy while minimizing systemic adverse effects, potentially enabling personalized approaches for intracranial hypertension and hydrocephalus management.

Recent pharmacological advances have expanded beyond traditional carbonic anhydrase inhibition toward selective membrane transport protein modulation. The NKCC1 inhibitor bumetanide demonstrates superior inhibitory stability compared to AZA (144, 145). While early preclinical studies questioned bumetanide’s efficacy in reducing CSF secretion, contemporary research has challenged these initial conclusions (168–170).

In IIH management, novel therapeutic strategies have emerged. Glucagon-like peptide-1 receptor agonists (GLP-1RAs) demonstrate efficacy in reducing CSF secretion and ICP (146, 147). A significant advancement involves AZD4017, an 11β-hydroxysteroid dehydrogenase type 1 (11β-HSD1) inhibitor. Randomized controlled trials validate its safety and efficacy in ICP reduction, with therapeutic effects correlating with decreased serum cortisol-to-cortisone ratios (148).

For tumor-associated hydrocephalus (TAH), Li et al. (149) identified novel therapeutic targets through single-nucleus RNA sequencing and spatial transcriptomics. Their research revealed CP mast cell-mediated disruption of epithelial cilia via the trypsin-PAR2-FoxJ1 pathway, augmenting CSF production. The BBB-permeable trypsin-like protease inhibitor BMS-262084 demonstrates effective TAH inhibition and amelioration of epithelial ciliary damage *in vivo*, establishing new paradigms for targeted TAH therapy.

#### 5.2.2 Modulating AQP4

Current therapeutic strategies targeting AQP4 encompass three primary approaches: gene silencing/activation, protein modification, and direct molecular interaction to modulate AQP4 quantity and polarization state (150). In genetic regulation, microRNA-130b demonstrates neuroprotective effects in ischemic conditions through AQP4 targeting (151, 152). Natural compounds, including apigenin and curcumin, attenuate post-hemorrhagic cerebral edema via downregulation of AQP4 gene and protein expression (153, 154). While AZA remains the most efficacious non-specific AQP4 expression inhibitor, the development of non-toxic AQP4-specific inhibitors continues to be an active area of investigation.

Recent advances have revealed novel therapeutic approaches. Liu et al. (155) employed spatial transcriptomics to demonstrate that moxibustion enhances AQP4 polarization in APP/PS1 mice through upregulation of hypothalamic Cox6a2 expression. Melatonin augments brain waste clearance by optimizing AQP4 polarization’s circadian rhythm (156). Additionally, acute AQP4 inhibition with TGN-020 reduces early-phase cerebral edema and promotes post-stroke recovery by attenuating peri-infarct astrocyte proliferation and AQP4 depolarization (55).

The identification of small molecules specifically modulating meningeal lymphatic drainage for Aβ clearance represents an innovative therapeutic approach for AD. Borneol enhances lymphatic vessel permeability and diameter, facilitating macromolecular transport to dCLNs. It promotes lymphatic clearance through upregulation of FOXC2, VEGFC, and LYVE-1 in the meninges, improving clearance of various macromolecules (2–45 kDa). Notably, borneol ameliorates cognitive deficits in Aβ-injected mice while reducing cerebral Aβ burden (157).

Angiotensin II type 1 (AT1) receptor modulation presents another therapeutic avenue. AT1 receptor deficiency preserves BBB integrity and prevents occludin and ZO-1 reduction following TBI. AT1-knockout mice demonstrate attenuated increases in APP expression and Aβ1-42/Aβ1-40 levels compared to wild-type counterparts post-TBI. Furthermore, AT1 receptor deficiency significantly inhibits TBI-induced AQP4 depolarization (158).

The AQP4 facilitator TGN-073 enhances glymphatic transport in normal rat brain, demonstrating increased Gd-DTPA distribution and elevated parenchymal uptake compared to vehicle controls. Enhanced water diffusivity in TGN-073-treated subjects indicates augmented water flux (159).

Intranasal oxytocin administration demonstrates multifaceted therapeutic effects through modulation of AQP4 polarization, cerebral hemodynamics, and meningeal lymphangiogenesis. This approach normalizes both glymphatic and meningeal lymphatic system function in murine models, facilitating efficient CSF drainage and neurotoxic waste clearance (160).

#### 5.2.3 Recombinant VEGF-C

Recombinant vascular endothelial growth factor C (VEGF-C) demonstrates significant therapeutic potential in enhancing meningeal lymphatic function. Research has established that compromised MLVs result in impaired CSF drainage and exacerbation of AD manifestations. VEGF-C administration activates VEGFR-3 signaling, promoting lymphatic endothelial cell proliferation and MLV development, thereby augmenting CSF drainage and clearance efficiency (161).

Recent investigations have identified Down syndrome critical region 1 (DSCR1) as a novel regulatory factor in MLV development. DSCR1 overexpression demonstrates significant improvement in meningeal lymphatic drainage in amyloid pathology murine models (162). In ischemic stroke studies, pretreatment with adeno-associated virus expressing mouse full-length VEGF-C (AAV-mVEGF-C) via intracerebroventricular administration enhances CSF drainage and improves stroke outcomes. Notably, these neuroprotective effects are abolished following cauterization of dCLNs afferent lymphatics (163).

In TBI research, Bolte et al. (164) demonstrated that mild TBI induces severe meningeal lymphatic drainage deficits, with morphological alterations persisting beyond 1 month post-injury. Pre-existing lymphatic dysfunction in TBI subjects correlates with enhanced neuroinflammation and cognitive deterioration. Significantly, VEGF-C-mediated restoration of meningeal lymphatic drainage function in aged TBI mice demonstrates efficacy in ameliorating TBI-induced neuroinflammatory responses.

#### 5.2.4 Emerging small molecule drugs

Yoda1, a selective Piezo1 channel agonist, demonstrates therapeutic efficacy in conditions characterized by elevated ICP and/or compromised CSF flow, such as craniosynostosis or age-related alterations. This small molecule reduces ICP and enhances CSF flow dynamics through mechanisms involving restoration of meningeal lymphangiogenesis, augmentation of dCLNs drainage, and optimization of brain-CSF perfusion (165).

Recent investigations indicate that ketoprofen and 9-cis retinoic acid (RA) maintain meningeal lymphatic wall integrity and promote lymphatic proliferation through upregulation of lymphatic vessel-specific proteins. These agents demonstrate multiple beneficial effects following TBI, including enhanced meningeal lymphatic function, improved CSF drainage, accelerated brain edema resolution, attenuated neuroinflammatory responses, and reduced reactive oxygen species (ROS) formation, collectively contributing to improved clinical outcomes (166).

Liu et al. (157) demonstrated that exogenous interleukin-33 (IL-33) administration enhances motor and cognitive performance in TBI mouse models. During the acute phase, IL-33 facilitates CSF-ISF exchange, reverses AQP-4 dysregulation and depolarization in cortical and hippocampal regions, enhances MLV drainage to dCLNs, and attenuates tau accumulation and glial activation.

## 6 Conclusions and future perspectives

The evolving understanding of CSF circulation systems has led to diversification of non-surgical therapeutic approaches, ranging from conventional pharmacological interventions to innovative neuromodulation strategies. However, the heterogeneous etiology among patients presents significant challenges, as single therapeutic modalities rarely address the comprehensive needs of all patients. This necessitates individualized treatment strategies that carefully consider patient-specific factors, including age, comorbidities, and pathophysiological characteristics.

The clinical implementation of novel therapeutic approaches faces multiple challenges. These include incompletely elucidated mechanisms of action, non-standardized efficacy assessment criteria, and limited long-term data from large-scale population studies. For instance, emerging interventions such as ta-VNS and NIBS demonstrate promising preliminary results in modulating glymphatic system function and enhancing CSF waste clearance. However, these approaches still require validation through large-scale clinical trials and long-term efficacy assessment. Furthermore, optimization of combination therapy strategies remains an important consideration, particularly regarding the selection of optimal therapeutic combinations for specific pathological conditions.

Future research directions should prioritize several key areas: First, deeper investigation of molecular mechanisms underlying CSF circulation regulation to provide theoretical foundations for novel targeted therapies; second, implementation of large-scale, multicenter clinical trials to establish standardized efficacy assessment protocols; and finally, development of personalized treatment decision support systems utilizing artificial intelligence technologies to achieve precision medicine objectives. Additionally, emphasis should be placed on strengthening translational research between basic science and clinical practice, promoting interdisciplinary collaboration, and fostering continuous innovation in CSF dynamic dysfunction therapeutics.
